# Endoscopic Hallmarks of Sessile Serrated Lesions: A Systematic Review of an Evolving Concept

**DOI:** 10.3390/medicina62061185

**Published:** 2026-06-18

**Authors:** Adrian Mită, Liviu Martin, Oana Criciotoiu, Iancu Emil Pleşea

**Affiliations:** 1 Department of Medical Semiology, University of Medicine and Pharmacy of Craiova, 200349 Craiova, Romania; 2 Department of Medical Assistance, Faculty of Medical Care, Titu Maiorescu University of Bucharest, 040051 Bucharest, Romania; 3 Department of Neurology, University of Medicine and Pharmacy of Craiova, 200349 Craiova, Romania; oana.criciotoiu@umfcv.ro; 4 Doctoral School, Carol Davila University of Medicine and Pharmacy, 050474 Bucharest, Romania; pie1956@yahoo.com; 5 Department of Pathology, Bagdasar—Arseni Emergency Clinical Hospital, 041915 Bucharest, Romania

**Keywords:** colorectal cancer, colon polyps, serrated adenomas, diagnosis, endoscopy, colonoscopy, systematic review

## Abstract

*Background and Objectives*: Sessile serrated lesions (SSLs) are increasingly recognized as relevant precursors in alternative pathways of colorectal carcinogenesis, yet their endoscopic recognition remains challenging. *Materials and Methods*: We conducted a systematic review of studies reporting endoscopic and patient-related features associated with SSLs. PubMed/MEDLINE was searched for studies published between 2003 and November 2025. Eleven studies including 13,453 participants were identified. *Results*: Frequently reported endoscopic hallmarks included proximal colon location, larger lesion size, flat or slightly elevated morphology, indistinct borders, mucous cap, cloud-like surface, and pit-pattern features consistent with Kudo/Fujii types III–IV. Considerable heterogeneity in definitions and analytical reporting was observed. *Conclusions*: Although several features may raise suspicion for SSLs, standardized evidence remains limited, supporting a cautious and comprehensive approach to polyp assessment.

## 1. Introduction

According to GLOBOCAN 2022 estimates, CRC accounted for approximately 1.93 million incident cases and 904,000 deaths globally, ranking among the leading causes of cancer incidence and mortality [1,2]. Beyond the classical adenoma-carcinoma sequence, multiple evolutionary pathways of colorectal carcinogenesis have been described, including the serrated pathway. Serrated colorectal lesions, particularly sessile serrated lesions (SSLs), are now recognized as clinically relevant precursors in this alternative pathway.

The classical adenoma-carcinoma sequence has long served as a foundational model for CRC development in both sporadic and hereditary settings, driven by stepwise accumulation of genetic and epigenetic alterations. [3]. At least two major carcinogenic pathways have been described, namely chromosomal instability and microsatellite instability; inherited syndromes such as familial adenomatous polyposis (FAP) and hereditary non-polyposis CRC (HNPCC) exemplify these mechanisms. Although there are different ways, they converge on common pathological entities that have extreme functions in the regulation of normal crypt homeostasis [4].

More recent genetic and pathological insights support the concept of multiple evolutionary routes to CRC beyond the conventional adenoma-carcinoma sequence. In particular, serrated lesions—including SSLs and traditional serrated adenomas—are key precursors in alternative carcinogenic pathways and may be under-recognized in clinical practice because they are subtle, inconspicuous, and often located in areas where complete mucosal inspection is technically demanding [5,6].

Hyperplastic polyps (HPs) are the most frequently encountered serrated colorectal polyps and have traditionally been considered non-neoplastic. However, accumulating evidence shows that a subset of hyperplastic or hyperplastic-like lesions may represent early steps within the serrated pathway, with potential progression to dysplastic serrated lesions and ultimately adenocarcinoma [7,8,9,10].

However, in the last two decades, a distinct alternative pathway—the serrated pathway—has been identified as a critical contributor to carcinogenesis. While originally believed to be benign, sessile serrated lesions (SSLs), along with traditional serrated adenomas (TSAs), are now recognized as precursors for a significant portion of colorectal cancers. It is estimated that up to 15–30% of all CRCs arise via this serrated pathway [10]. SSLs are particularly relevant to endoscopists because they are often flat or slightly elevated, pale, covered by adherent mucus, and have poorly demarcated borders. These features contribute to missed lesions and interval CRC, especially in the proximal colon [8,9].

Colonoscopy plays a central role in CRC prevention by enabling detection and removal of precancerous lesions. Nonetheless, prevention may be compromised by practical and operator-dependent factors, including inadequate bowel preparation, missed right-sided lesions, limited withdrawal time, variable endoscopic expertise, and insufficient familiarity with serrated morphology. Multiple studies have attempted to describe endoscopic features suggestive of SSLs; however, reports are heterogeneous and have not been consistently synthesized.

### Objective

To systematically review the literature on patient and polyp-related endoscopic features reported to be associated with sessile serrated adenomas/polyps (SSLs), in order to facilitate their recognition as precancerous lesions in routine practice.

## 2. Materials and Methods

### 2.1. Reporting Guidelines and Registration

This systematic review was conducted and reported in accordance with the Preferred Reporting Items for Systematic Reviews and Meta-Analyses (PRISMA) 2020 statement [11]. A completed PRISMA 2020 checklist is provided as Supplementary File S1. The review protocol was not prospectively registered in PROSPERO or another publicly accessible registry.

### 2.2. Search Strategy

MEDLINE (via PubMed) was searched for studies published from 1 January 2003 to 30 November 2025 investigating endoscopic features associated with sessile serrated lesions (SSLs). The search combined terms related to serrated colorectal lesions, colon polyps, and colonoscopy. The detailed search string was as follows:

((“intestinal polyps”[MeSH Terms] OR “colonic polyps”[Title/Abstract] OR polyp*[Title/Abstract]) AND (“colonoscopy”[MeSH Terms] OR colonoscop*[Title/Abstract]) AND (serrated[Title/Abstract] OR “sessile serrated lesion*”[Title/Abstract] OR “sessile serrated adenoma*”[Title/Abstract] OR “sessile serrated polyp*”[Title/Abstract] OR “serrated adenoma*”[Title/Abstract] OR “serrated polyp*”[Title/Abstract] OR SSA/P[Title/Abstract] OR SSP[Title/Abstract])). Language restrictions were applied to English, French, Spanish, and Portuguese. In addition, the reference lists of all eligible studies and relevant reviews were manually screened to identify additional records.

### 2.3. Eligibility Criteria

Eligible studies included cohort, cross-sectional, case-control, case-series, and randomized studies reporting endoscopic features associated with SSLs. Studies were included if they fulfilled the following criteria: (a) evaluation of endoscopic and/or clinical variables potentially associated with SSLs; (b) an eligible observational or interventional study design; and (c) reporting of lesion-level or patient-level features relevant to SSL recognition. Reviews, editorials, purely pathological studies without endoscopic descriptors, studies focused on inflammatory bowel disease, and articles addressing medical or surgical treatment of CRC rather than endoscopic recognition were excluded.

### 2.4. Study Selection

**Figure 1:**
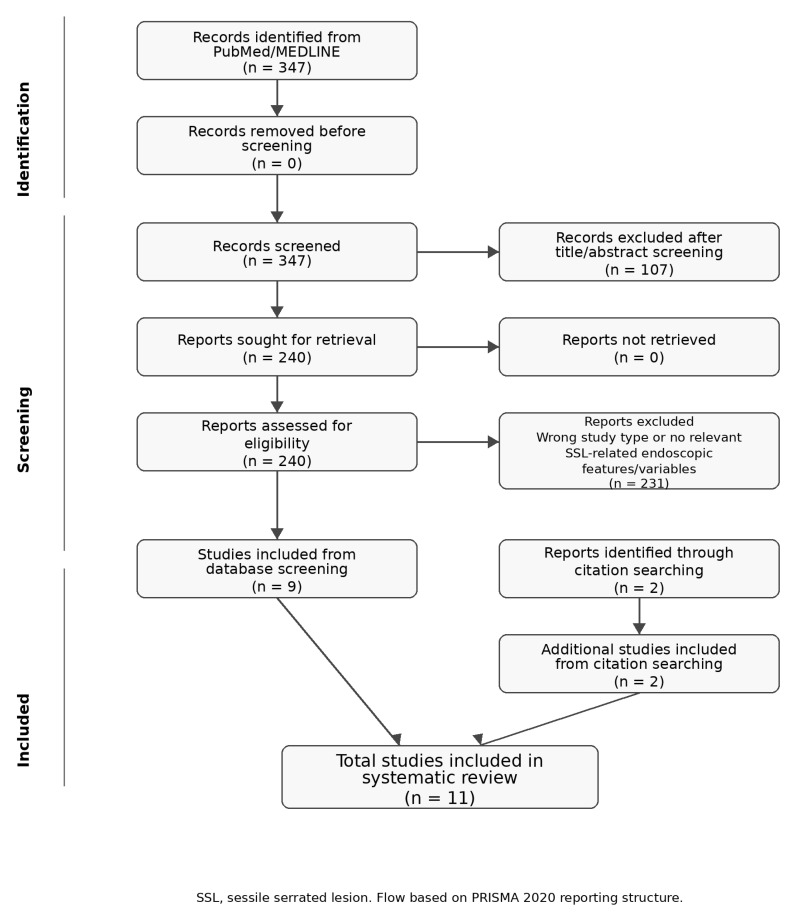
PRISMA 2020 study selection flow diagram.

After screening, studies focused purely on colorectal cancer and sessile serrated lesions (SSLs). Following title and abstract screening, studies focused on predicted markers alone, inflammatory bowel disease, diverticulosis, medical or surgical treatment of colorectal cancer, and purely pathological analyses were excluded. Full-text assessment was subsequently performed for the remaining articles, and any disagreements were resolved by expert consensus. The database search identified 347 records. After title and abstract screening, 107 records were excluded. The full texts of the remaining 240 reports were assessed for eligibility, with no reports unavailable for retrieval. Of these, 231 full-text reports were excluded mainly because they were of the wrong study type or did not report relevant SSL-related endoscopic features or variables. Consequently, 9 studies met the eligibility criteria through database screening. Finally, 2 additional eligible studies were identified at the end of the process through iterative screening of the reference lists of included articles and relevant reviews. This resulted in a total of 11 included studies, as provided in the PRISMA flow diagram (Figure 1). At each stage, disagreements were resolved by expert consensus.

### 2.5. Data Extraction

Articles extracted data included study characteristics, patient demographics, polyp descriptors, and reported associations. Once article selection was completed, the following data were collected from each article:

(a) Article identification: title, author(s) and publication date;
(b) Methods and variables: study design; participant selection methods (sample size, setting, selected versus consecutive samples, age, sex, and family history when available); exclusion criteria; endoscopic procedure characteristics (type of scope, bowel preparation quality, number of endoscopists, and training level when reported); polyp descriptors, including location (proximal or distal to the splenic flexure, as reported in most studies), number, size (converted to millimeters), shape (Paris classification when available), contour, surface, pit pattern (Kudo and/or Fujii classifications when reported) [12,13], vascular pattern, reported association measures or statistical significance, and potential bias domains addressed by QUADAS.

Studies were considered prospective or retrospective according to the moment at which lesion classification was assigned relative to histological outcome assessment. Studies were classified as cohort studies when association measures were reported and as case series when they provided descriptive lesion features without association testing (Table 1).

### 2.6. Quality Assessment

Study quality was assessed using the QUADAS checklist [25]. The assessment was performed by Liviu Martin (L.M.), one review author; therefore, potential subjective bias cannot be excluded. The QUADAS tool includes 14 items scored as yes/no/unclear. To improve transparency, both the overall score and the distribution of key domain-level concerns were reported, rather than relying on the summary score alone.

The authors state that no major interventions of the Generative Artificial Intelligence (GenAI) mode were necessary in this work. The use of GenAI was only sporadically used for superficial text editing.

### 2.7. Synthesis Methods

Because the included studies were heterogeneous with respect to design, comparator lesions, lesion definitions, endoscopic descriptors, imaging modalities, and statistical reporting, a formal meta-analysis was not planned. Findings were synthesized qualitatively by grouping the reported descriptors into clinically relevant domains: location and size; shape and contour; surface-related features; and pit-pattern and vascular features. When available, association measures, diagnostic accuracy indices, and statistical significance values were extracted and reported as presented in the original studies. Formal assessments of reporting bias, certainty of evidence, and sensitivity analyses were not performed because quantitative synthesis was not conducted and the small, heterogeneous evidence base did not permit meaningful assessment.

## 3. Results

Eleven studies met the inclusion criteria, with a mean QUADAS score of 9.5. Although the overall QUADAS scores were relatively high, several recurring methodological issues were identified across the included studies. These mainly involved selected rather than consecutive sampling, heterogeneous comparator groups, and inconsistent reporting of uninterpretable findings or withdrawals. Therefore, QUADAS scores should be interpreted together with the domain-level summary rather than as stand-alone measures of study reliability (Supplementary Tables S1 and S2).

The main recurring concerns were non-consecutive or selected sampling, limited reporting of blinding, incomplete reporting of diagnostic accuracy measures, and heterogeneous comparator groups. These issues were not sufficient to exclude the studies, but they weaken the certainty with which any single endoscopic feature can be interpreted as diagnostic.

Reported descriptors included lesion location, size, morphology, contour, surface characteristics, pit-pattern features, and vascular patterns (Table 2). To provide a structured comparison despite the absence of meta-analysis, findings were grouped into four clinically relevant domains: (i) location and size; (ii) shape and contour; (iii) surface-related features; and (iv) pit-pattern and vascular features.

Across these domains, SSLs were most often described as proximal, larger than comparator hyperplastic lesions, flat or slightly elevated, and associated with indistinct borders, mucous cap, rim debris/bubbles, cloud-like surface, and selected pit-pattern or vascular findings. However, not all studies evaluated the same descriptors, and several findings were reported only in single studies.

### 3.1. Description of Studies

We have included studies (*n* = 11) evaluating endoscopic features and/or the association of specific variables with SSLs. Studies were considered as prospective or retrospective by taking into account the moment at which adenomas classification was selected. Since in all articles all subjects conducting endoscopy or presenting adenomas or suspected serrated lesion, studies were classified as cohort if association measures were reported or case series if they were not. According to these classification six prospective cohorts, two prospective case series and three retrospective cohorts were included. The assessment through QUADAS checklist led to a mean score of 9.5 present items, ranging from 8 to 11.

### 3.2. Participants’ Characterization

Regarding sample size of the included studies, two studies enrolled 2309 [16] and 10,199 [15] subjects respectively. These two studies are of major importance since they represent data from a total of 16 multiple centres’ and in addition from areas with high incidence of colon cancer. In the other hand, some studies can be considered as specific since they have selected the included cases by excluding those patients with other comorbidities. The total number of participants enrolled in the SR reached 13,453.

Participants’ selection includes both “selected” (n = 7) and “consecutive” (n = 4) samples. The majority of studies was conducted in a single centre, except for 2 studies [15,17]. Most patients attending the health centres/hospitals were complaining of gastro-intestinal symptoms or came for screening/surveillance. One study [19] enrolled only asymptomatic patients.

Age was assessed in ten of our studies. Some studies have excluded subjects with less than 18 years [16,18] or 20 years [15]. Although the age highly varied, the presence of a high occurrence of SSLs was found around the sixth decade. However, some authors have excluded patients with polyps larger than 1 cm [23] and those with HNPCC or FAP [16,19,23].

By gender, we found that 9 of the studies reported a predominance of males with a rate of 60%. One article [21] included only male subjects, while Singh [17] excluded pregnant or lactating women. A case series article included 2309 subjects being 46% male [16] and one cohort study reported 52% of males in the 10,199 participants [15].

Overall exclusion criteria of patients in these studies were: IBD; those with anticoagulant therapy; the presence of juvenile polyps; hyperplastic and TSAs [22]; age under 18 years or poor bowel preparation. In addition, the presence of a history of CRC/FAP led to subjects’ exclusion in 3 manuscripts.

In what concerns histological outcomes, it was reported in 7 studies that pathologists were not aware of the results of endoscopic imaging and also in one study [22], it was clearly reported that gastroenterologists were blind to polyp’s histology.

Endoscopic features and assessed variables for SSLs obtained from the included studies are described in Table 2 and Table 3.

All the included studies described the polyps’ location found by endoscopy. Five studies reported prevalence in the proximal and five in the distal part of the splenic flexure. An article considered only the proximal sigmoid region. Considering the number of articles, the distal part of splenic flexure location was more frequent (54% of articles). However, the largest study (including 76% of the total enrolled patients) [15], reported a predominance of polyps in the proximal colon. The only study assessing an association between location and SSLs [14] concluded that 85% were on the right side which is a significantly higher proportion when compared with TSAs and HPs (*p* < 0.001).

During endoscopy, in the included studies, SSLs location was equally distributed both in the proximal and in the distal part of the colon.

In terms of size, it should be noted that some studies have excluded polyps larger than 1 cm [23]. Others have examined only pre-sized polyps, below par (under 1 cm) [17,19]. Results draw attention that at sizes exceeding 6 mm the SSLs predominate in the proximal colon, describing only one study with values lower than 5 mm for this area [19]. And in the distal colon, they are predominantly smaller, starting from only 3 mm [17,18,21,23]. In the only study assessing this variable association, authors concluded that SSLs were significantly larger when compared with TSAs and HPs (SSA/P vs. HP *p* < 0.001; SSA/P vs. TSA *p* < 0.01) according to Ishigooka.

Shapes as flat, elevated and protruded were all described for the proximal lesions, with the flat being predominantly described in four articles [13,16,18,20]. In opposition Hiraoka reported a predominance of a protruded shape [15]. In the distal part, were observed also flat, elevated, sessile and non-polyposis shapes, all with a relatively equal proportion. Flat or elevated shapes are the most common overall, with a slightly predominance of the flat ones in the right side of the colon. In the only article that compares the shape of SSLs with TSAs and HPs it was concluded that the first presented more frequently a flat surface when compared with the last two (*p* < 0.001) [14].

Three cases described the contour as irregular. One of them [24] was saying that it is indistinct because of vague demarcation of borders. An article [22] mentioned that 37.3% of polyps had an irregular contour which was associated with dome shape (OR 3.66, *p* = 0.002). Only Kim described it as regular, but with no accuracy of distinguishing SSA/Ps through this feature.

The surface of polyps was scarcely described. In 3 studies describing this characteristic a flat [14], round/oval [21] and cloud-like [24] were reported. The main conclusion of a study was that significantly more SSLs were identified as flat surface, presenting abundant mucin and a whitish change in colour when compared with TSAs and HPs (with all presenting a *p* < 0.001) [14]. However, another study concluded that SSLs presented less mucin (OR [odds ratio] 0.04, *p* = 0.0001) [22].

One study using multivariate analysis demonstrated that presence of an indistinctive borders (OR 3.11), with accuracy of 73%, and cloud-like surface (OR 2.65), with accuracy of 68%, were two independent predictive features of SSA/Ps on high resolution-white light endoscopy (HR-WLE) [24].

Under Narrow Band Imaging (NBI) a cloud-like surface (OR 4.91), with accuracy of 76%; an irregular shape (OR 3.17), with accuracy of 74%; indistinctive borders (OR 2.38), with accuracy of 71%; and dark spots inside the crypts (OR 2.05), with accuracy of 65%, were found to be independent predictors of SSA/Ps.

For the mucosal pattern, in two cases, the authors used Kudo’s classification or Kudo’s plus Fujii’s clinical classification [12,13,19,20]. Thereby, for proximal polyps (<10 mm, flat and/or elevated) were showed pit patterns III/IV/V according to Kudo’s classification [12], and large, elongated pits (III) or branched/gyrus-like pits (IV) according to Fujii’s classification [13].

One author found significant association between the presence of mucous cap and rim of debris or bubbles (OR 3.8, *p* = 0.002) as well as for mucous cap obscuring the underlying vessels (OR 3.2, *p* < 0.003) [22].

As described in a recent study, the mucosal pattern for Singh was defined as circular/oval/linear/cerebriform pits (central white configuration with dark vessels bordering the pits) [28]. Two articles [21,23] described them as round/oval or tubulo-gyrus pattern.

For the vascular pattern, one study described the presence of dark vessels bordering the pits [16], and another the interruption of the underlying mucosal vascular pattern for SSA/Ps comparing with the red colour found in APs [22].

A formal meta-analysis was not performed because the included studies were highly heterogeneous with respect to design, comparator lesions, lesion definitions, endoscopic descriptors, imaging modalities, and statistical reporting. Many studies did not provide effect estimates suitable for pooling. Instead, a structured qualitative synthesis was performed by grouping findings into clinically meaningful domains and distinguishing repeatedly reported features from single-study observations.

## 4. Discussion

This systematic review shows that endoscopic recognition of SSLs relies on a constellation of subtle findings rather than on a single pathognomonic feature. Across the 11 included studies, the most frequently reported descriptors were proximal location, larger lesion size, flat or slightly elevated morphology, indistinct borders, mucous cap or adherent debris, cloud-like surface, and pit-pattern abnormalities suggestive of serrated histology [14,15,16,17,18,19,20,21,22,23,24]. The strength of these conclusions is limited by heterogeneous definitions, non-uniform comparator groups, and inconsistent reporting of diagnostic performance measures.

In the current state of the art, SSLs should be interpreted within the broader category of non-polypoid and subtle colorectal neoplasia. Facciorusso et al. emphasized that flat or slightly elevated colorectal lesions are less conspicuous than polypoid lesions, may be missed by inexperienced endoscopists, and often require deliberate optical assessment using careful morphology, pit-pattern, and vascular-pattern evaluation to guide treatment [29]. This framework is directly relevant to SSLs, which are frequently pale, minimally elevated, mucus-covered, and located in the proximal colon.

The state of the art is also influenced by the evolving histopathologic and molecular definition of serrated lesions. Distinguishing SSLs from HPs is not always straightforward, and reproducibility depends on the recognition of architectural abnormalities, particularly at the crypt base [30,31]. Molecularly, serrated carcinogenesis is commonly associated with BRAF mutation, CpG island methylator phenotype, and, in a subset of cases, microsatellite instability, whereas conventional CRC more often follows APC/KRAS/TP53-driven adenoma-carcinoma progression and chromosomal instability [31,32]. Additional molecular observations, such as somatic CCK2R mutations that increase receptor activity and promote oncogenic phenotypes, and chromatid-cohesion defects that may contribute to chromosomal instability in CRC, illustrate the broader mutational heterogeneity of colorectal tumors [33,34].

Although theranostic approaches are not yet part of routine SSL recognition, they represent an emerging state-of-the-art field in colorectal oncology. Radiolabeled vitamins and nanosystems, including nanoparticles, are being explored as strategies that integrate molecular imaging with targeted therapy [35]. Preclinical work has also evaluated 68Ga-labeled radiopharmaceuticals in cancer models overexpressing CCK2R, using biodistribution and radiomics analyses to assess target engagement [36]. These approaches remain investigational for CRC and are not substitutes for high-quality colonoscopy, but they contextualize the growing interest in linking molecular biomarkers with imaging and treatment.

The quality assessment adds an important cautionary layer. QUADAS was appropriate because the review question concerns diagnostic recognition, but the fact that the assessment was performed by a single reviewer is a methodological limitation. In future updates, two independent reviewers should ideally perform quality assessment with a consensus process. In the present review, the item-level and domain-level summaries show that the main concerns involved patient selection, blinding, and incomplete diagnostic accuracy reporting, which may inflate or obscure the apparent usefulness of individual features.

The structured comparison across studies suggests that location and size are useful contextual clues rather than diagnostic criteria. Although the largest dataset and the comparative analysis by Ishigooka et al. support a predominance of proximal lesions [14,15], several studies also reported distal SSLs or small lesions [16,17,18,21,23]. Therefore, the practical implication is not that SSLs are exclusively right-sided, but that right-sided flat or mucus-covered lesions deserve heightened attention, while small distal serrated lesions should not be dismissed automatically.

Morphology, contour, and surface features appear more directly useful for real-time suspicion. Flat or slightly elevated morphology was repeatedly reported, but protruded, sessile, and non-polypoid variants were also described [14,15,16,19,22,24]. Indistinct borders, cloud-like surface, mucous cap, rim debris/bubbles, and obscuration of the underlying vascular pattern were among the most clinically actionable findings, particularly in the studies by Hazewinkel et al. and Tadepalli et al. [22,24]. These signs should be interpreted together, because none is sufficiently specific in isolation.

A slightly elevated flat lesion with wide pits has also been described at the margin of laterally spreading tumors and referred to as a “skirt” [37]. Although this entity is not synonymous with SSL, it reinforces the broader message that subtle marginal architecture, crypt pattern, and surface morphology may signal biologically relevant colorectal neoplasia and should prompt careful delineation before resection.

Pit-pattern and vascular descriptors were reported less consistently, but remain relevant when magnification or image-enhanced endoscopy is available. Studies using Kudo- and Fujii-based approaches described large, elongated, branched, or gyrus-like pit patterns in suspected SSLs [12,13,19,20,38], whereas other authors reported circular, oval, linear, cerebriform, or tubulogyrus patterns [17,21,28]. Vascular features were even less standardized, ranging from dark vessels bordering pits to interruption or obscuration of the background vascular pattern [16,22].

Image No 1. Examples of Kudo’s classification scale of Polyps ranging from I through V [12,38].

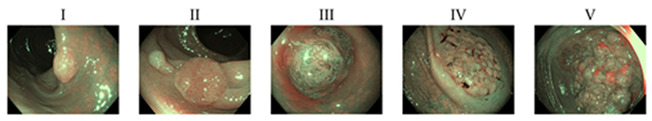

Legend: Classification of polyp mucosal pit patterns according to Kudo’s scale. Panels I and II represent non-neoplastic/benign patterns, panels III and IV demonstrate adenomatous structures, and panel V indicates a high-risk or invasive neoplastic pattern.

Taken together, these observations suggest that high-definition white-light endoscopy, chromoendoscopy, and virtual chromoendoscopy may improve confidence in lesion characterization, but the current evidence still does not support a universally adopted pit-pattern or vascular classification dedicated specifically to SSLs.

The clinical relevance of these findings extends beyond lesion recognition alone. SSLs are accepted precursor lesions within the serrated pathway of colorectal carcinogenesis [6,7,10]. From a practical perspective, subtle serrated lesions should not be underestimated during colonoscopy. Once suspected, careful delineation and complete resection are essential, particularly for proximal and non-diminutive lesions, because incomplete recognition or incomplete removal may undermine the preventive value of colonoscopy [8,29].

The absence of pooled estimates remains a limitation. However, a quantitative synthesis would have been unreliable because descriptors, thresholds, comparator groups, and imaging modalities differed substantially between studies. For this reason, the review provides a structured qualitative comparison rather than a meta-analysis. This approach allows clinically useful patterns to be summarized while avoiding a misleading numerical estimate from non-comparable data.

This review has several strengths, including a focused clinical question, extraction of patient-related and lesion-related features, and synthesis of findings across different endoscopic modalities. Important limitations must also be acknowledged: database coverage was restricted to PubMed/MEDLINE; the quality assessment was performed by a single reviewer; only 11 studies met the eligibility criteria; and many studies were descriptive, retrospective, or incompletely reported. These limitations mean that the proposed hallmarks should be interpreted as suspicion-raising features rather than validated diagnostic criteria.

Future research should move beyond descriptive reporting and focus on prospective validation using harmonized endoscopic descriptors and uniform histopathologic criteria. Studies should report sensitivity, specificity, predictive values, interobserver agreement, and the incremental value of image-enhanced endoscopy. Integration of high-definition colonoscopy, virtual chromoendoscopy, artificial intelligence-assisted detection, and expert pathology review may help define reproducible models for SSL recognition. Until such evidence becomes available, detection should rely on meticulous mucosal inspection, recognition of clusters of subtle features, and complete resection of suspicious lesions.

## 5. Conclusions

In summary, we would like to state that, our study was able to highlight some important features gathered together above (Table 3) that should be taken into consideration when performing a colonoscopy. However, many questions still remain about them and those other features mentioned so far in order to define an endoscopic profile of serrated lesions.

Several endoscopic features may raise suspicion for SSLs; however, no single hallmark is diagnostic. Until standardized and validated criteria become available, all detected colorectal polyps should be approached as potentially premalignant and resected completely.

## Figures and Tables

**Table 1:** Characterization and classification of the studies according to study type, QUADAS score and sample size.

Authors	Series Size (No)	Age (Mean)	Male Gender (%)	Polyps (n)	Selection	Setting	Exclusion Criteria	Outcome Histology	QUADAS Quality #
Prospective cohort									
Ishigooka et al., 2012 [14]	-	61	74	118	Selected cases	Single center	Regular As and juvenile Ps were excluded (6 cases)	23 HPs 50 SSAs/Ps 39 TSAs	11
Hiraoka et al., 2010 [15]	10,199	59	52	-	Selected cases	Multicenter	*	**	10
Rondagh et al., 2011 [16]	2309	58	46	493	Consecutive sample	Outpatients Single center	Patients with Hereditary form of CRC Age <18 years	493 SSAs/Ps ##	10
Singh et al., 2012 [17]	33	62	36	50	Consecutive sample	Multicenter	Pregnant, lactating, or severe comorbidities	31 HPs 19 SAs/Ps	9
Boparai et al., 2010 [18]	22	60	55	209	Consecutive sample	Single center	IBD, Severe coagulopathy, Age < 18 years, Poor bowel preparation Known germ line APC mutation or bi-allelic *MYH*	116 HPs 42SSAs/Ps 24 As	9
Emura et al., 2006 [19]	170	69	88	264	Selected cases	Single center	History of CRC, Colonic surgery FAP IBD Anticoagulation therapy	-	8
Prospective case series									
Sonwalkar et al., 2006 [20]	476	66	54	709	Selected cases	Single Center	-	163 SSAs/Ps ###	9
Rastogi et al., 2009 [21]	40	62	100	65	Selected cases	Single center	-	27 HPs 38 SAs/Ps	9
Retrospective cohort									
Tadepalli et al., 2011 [22]	124	62	35	158	Consecutive sample	Outpatients	HPs TSAs	158 SSPs	10
Kim et al., 2008 [23]	35	-	-	50	Selected cases	Single Center	Polyp’s ≥ 1 cm Other Ps than APs/HPs	17 HPs 33 SAs	10
Hazewinkel et al., 2013 [24]	45	61	-	150	Selected cases	Single tertiary referral centre	-	50 SSAs/Ps 50 HPs 50 As	9

Legend: As, adenomatous; APC, adenomatous polyposis coli gene; CRC, colorectal cancer; FAP, familial adenomatous polyposis; HPs, hyperplastic polyps; IBD, inflammatory bowel disease; MYH, MutY human homologue gene; SAs/Ps, serrated adenomas/polyps; TSAs, traditional serrated adenomas; * Cases ≤ 20 years of age, with prior resection of any part of the colon, with FAP or hereditary nonpolyposis colorectal cancer, with IBD, and whose data lacked clinical in-formation or histologic information on polyps; ** 7112 No adenoma, 1514 Adenoma ≤ 9 mm, 1573 Advanced neoplasia, 450 Adenoma ≥ 10 mm, 216 Tubulovillous or villous adenoma, 199 High-grade dysplasia, 708 Cancer, 140 LSPs; # Number of positive answers to QUADAS checklist [25]; ## According to “WHO classification of tumours of the digestive system” [26]; ### According to “The Vienna classification of gastrointestinal epithelial neoplasia” [27].

**Table 2:** Included studies and endoscopic descriptors evaluated.

Study	Design	Location	Size (mm)	Shape	Contour	Surface	Pit Pattern	Vascular Pattern	Clinical Implication	Limitations/Notes
Ishigooka et al., 2012 [14]	Prospective cohort	Proximal	>6	Flat	-	Flat	-	-	Flat proximal lesions deserve careful inspection (often subtle).	Limited descriptor set; no pit/vascular detail reported.
Hiraoka et al., 2010 [15]	Prospective cohort	Proximal	≥10	Protruded	Irregular	-	-	-	Large proximal serrated lesions may appear protruded and irregular, not only flat.	Few surface/pattern descriptors; limited optical guidance.
Rondagh et al., 2011 [16]	Prospective cohort	Distal	≥6	Non-polypoid	-	-	-	-	Distal serrated lesions can be non-polypoid, increasing the risk of missed detection.	Minimal description beyond size/location.
Singh et al., 2012 [17]	Prospective cohort	Distal	>3	-	-	-	Circular/oval/linear/cerebriform pits	Dark vessels bordering pits	Enhanced imaging may support optical suspicion in diminutive distal lesions.	Pattern descriptors are not universally used; interobserver variability likely.
Boparai et al., 2010 [18]	Prospective cohort	Distal	>3	Flat	-	-	-	-	Even small, flat distal lesions may represent SSA/P and should not be dismissed as insignificant.	Lack of surface/pattern descriptors limits bedside optical rules.
Emura et al., 2006 [19]	Prospective cohort	Proximal	≤5	Flat/elevated	-	-	Kudo III/IV; Fujii: III–IV	-	Magnification/chromo pit patterns can help distinguish serrated lesions from innocuous polyps.	Technique-dependent; not always available in routine practice.
Rastogi et al., 2009 [21]	Prospective case series	Distal	>3	-	-	Round/oval	Round/oval; tubulogyrus	-	Surface and pit-pattern recognition may aid optical triage.	Case-series design; limited generalizability; unclear association testing.
Tadepalli et al., 2011 [22]	Retrospective case-control	Proximal	≥6	Flat	Irregular	Nodular (±mucus)	Mucous cap; rim debris/bubbles	Vascular pattern obscuration	A mucous cap and rim debris/bubbles are practical red flags; vascular obscuration may be supportive.	Retrospective design; selection bias possible; mucus findings are inconsistent across studies.
Kim et al., 2008 [23]	Retrospective case-control	Distal	>3	Elevated	Linear/regular	-	Tubular or gyrus-like	-	Serrated lesions are not always flat; some may be elevated with a relatively regular contour.	Contour finding contrasts with other reports; limited diagnostic accuracy reported.
Hazewinkel et al., 2013 [24]	Retrospective case-control	Proximal	≥5	Sessile/flat	Indistinct borders	Cloud-like	Dark spots in crypts	-	Indistinct borders and cloud-like surface are useful cues; NBI ‘dark spots’ can increase suspicion.	Requires training; not all studies evaluated these predictors; modality-dependent performance.

Abbreviations: Kudo’s classification and Fujii’s clinical classification; NBI, narrow-band imaging; SSA/P, sessile serrated adenoma/polyp; WLE, white-light endoscopy; “-” this study did not measured this variable. Was considered as distal or proximal to the splenic flexure.

**Table 3:** Practical endoscopic hallmarks of SSA/Ps (appearance, implications, limitations).

Hallmark (SSA/P)	Typical Endoscopic Appearance	Practical Clinical Implication	Key Limitations/Pitfalls
Proximal location	Often right-sided/proximal	Increase vigilance in the proximal colon; allocate adequate withdrawal time.	Location alone is not diagnostic; proximal lesions are easier to miss.
Larger size (especially proximal)	Frequently >5 mm proximally; can be small distally (>3 mm)	Do not dismiss small distal polyps; scrutinize larger proximal flat lesions.	Size thresholds differ across studies and are not standardized.
Flat or slightly elevated morphology	Often flat; sometimes slightly protruded	Actively search for subtle, carpet-like lesions; consider enhanced imaging if uncertain.	Morphology is variable; some serrated lesions can be protruded/elevated.
Irregular or indistinct borders/contour	Irregular outline; vague demarcation	Indistinct edges should prompt careful margin assessment before resection.	Some studies describe relatively regular contours; limited discriminative value.
Mucous cap/rim of debris or bubbles	Adherent mucus; debris/bubbles; may obscure vessels	Practical red flag on WLE; wash to reveal surface architecture and reassess.	Not universally present; mucus-related findings are inconsistent across studies.
Cloud-like/nodular/round-oval surface	Cloud-like surface or nodularity; round/oval flat surface	Cue to switch to enhanced imaging and document thoroughly.	Descriptors are subjective; interobserver variability likely.
Pit-pattern features (Kudo/Fujii III–IV)	Large elongated (III) or gyrus-like/branched (IV); tubulogyrus/cerebriform terms	Supports optical suspicion (magnification/chromo) and may help differentiate from HP.	Technique-dependent; classification use is inconsistent across centers.
NBI supportive signs	Dark spots in crypts; dark vessels bordering pits; vascular pattern interruption/obscuration	May increase confidence to treat as premalignant and resect completely.	Few studies assess vascular features; evidence remains limited and heterogeneous.

Abbreviations: HP, hyperplastic polyp; NBI, narrow-band imaging; SSA/P, sessile serrated adenoma/polyp; WLE, white-light endoscopy.

## Data Availability

Data are contained within the article.

## Supplementary Materials

The following supporting information can be downloaded at: https://www.mdpi.com/article/10.3390/medicina62061185/s1, Supplementary File S1. PRISMA 2020 Checklist; Supplementary File S2: Table S1. Domain-level summary of recurrent QUADAS-related concerns. Table S2. QUADAS Assessment of Included Studies.
