# Supplementary material for: Endoscopic Hallmarks of Sessile Serrated Lesions: A Systematic Review of an Evolving Concept
Source: Medicina (Kaunas). 2026 Jun 18;62(6):1185. doi: 10.3390/medicina62061185 (PMC13303899; doi:10.3390/medicina62061185)
Supplement: Supplementary file 1 [file medicina-62-01185-s001.zip › medicina-4367128-supplementary-file S1.pdf]

Supplementary File S1. PRISMA 2020 Checklist

Manuscript: Endoscopic Hallmarks of Sessile Serrated Lesions: A Systematic Review of an Evolving Concept

Note. Locations refer to the revised manuscript sections. Items that are not applicable are marked N/A with a brief justification.

Section	Item	Topic	Checklist item	Location in revised manuscript
TITLE	1	Title	Identify the report as a systematic review.	Title page
ABSTRACT	2	Abstract	Report the review using the PRISMA 2020 abstract items.	Abstract
INTRODUCTION	3	Rationale	Describe the rationale for the review in the context of existing knowledge.	Introduction
INTRODUCTION	4	Objectives	Provide an explicit statement of the objective(s) or review question(s).	Introduction, Objective
METHODS	5	Eligibility criteria	Specify inclusion and exclusion criteria and how studies were grouped for synthesis.	Methods, Eligibility Criteria
METHODS	6	Information sources	Specify all databases, registers, websites, organisations, reference lists, and dates searched.	Methods, Search Strategy
METHODS	7	Search strategy	Present the full search strategy for all databases/registers/other sources.	Methods, Search Strategy
METHODS	8	Selection process	Describe how records and reports were screened, how eligibility was assessed, and how disagreements were resolved.	Methods, Study Selection; Figure 1
METHODS	9	Data collection process	Describe how data were collected from reports and whether processes were independent or verified.	Methods, Data Extraction
METHODS	10a	Data items: outcomes	List and define the outcomes or result domains for which data were sought.	Methods, Data Extraction; Tables 1–3
METHODS	10b	Data items: other variables	List and define other variables collected and any assumptions or simplifications made.	Methods, Data Extraction; Tables 1–3
METHODS	11	Study risk of bias assessment	Describe methods used to assess risk of bias or methodological quality of included studies.	Methods, Quality Assessment; Results; Supplementary Tables S1–S2
METHODS	12	Effect measures	Specify the effect measures or association measures extracted or planned for each outcome.	Methods, Data Extraction; Methods, Synthesis Methods
METHODS	13a	Synthesis eligibility	Describe how studies were judged eligible for each synthesis.	Methods, Synthesis Methods
METHODS	13b	Data preparation	Describe data preparation for presentation or synthesis, including transformations where relevant.	Methods, Data Extraction; Methods, Synthesis Methods
METHODS	13c	Tabulation/display methods	Describe methods used to tabulate or visually display individual-study and synthesis results.	Methods, Synthesis Methods; Tables 1–3; Figure 1
METHODS	13d	Synthesis methods	Describe synthesis methods and provide a rationale for qualitative or quantitative synthesis choices.	Methods, Synthesis Methods; Results
METHODS	13e	Heterogeneity exploration	Describe methods used to explore possible causes of heterogeneity among study results.	Methods, Synthesis Methods; Discussion
METHODS	13f	Sensitivity analyses	Describe any sensitivity analyses used to assess robustness of synthesized results.	N/A — no quantitative synthesis was performed
METHODS	14	Reporting bias assessment	Describe methods used to assess risk of bias due to missing results in a synthesis.	N/A — formal assessment was not feasible for the qualitative synthesis
METHODS	15	Certainty assessment	Describe methods used to assess certainty or confidence in the body of evidence.	N/A — formal certainty grading was not performed
RESULTS	16a	Study selection: flow	Describe results of the search and selection process, ideally using a flow diagram.	Methods, Study Selection; Figure 1
RESULTS	16b	Study selection: excluded studies	Cite studies that appeared to meet inclusion criteria but were excluded, and explain why.	Methods, Study Selection; Figure 1; summarized exclusion reasons
RESULTS	17	Study characteristics	Cite each included study and present its characteristics.	Results; Table 1
RESULTS	18	Risk of bias in studies	Present assessments of risk of bias or methodological quality for included studies.	Results; Quality assessment paragraph; Supplementary Tables S1–S2
RESULTS	19	Results of individual studies	Present results for individual studies, including summary statistics or effect estimates where available.	Results; Tables 2–3
RESULTS	20a	Results of syntheses: included studies	For each synthesis, summarize characteristics and risk of bias among contributing studies.	Results; Tables 1–3
RESULTS	20b	Results of statistical syntheses	Present results of all statistical syntheses, including uncertainty and heterogeneity where applicable.	N/A — no meta-analysis was performed; qualitative synthesis reported in Results
RESULTS	20c	Heterogeneity results	Present results of investigations of possible causes of heterogeneity.	Discussion; qualitative heterogeneity described
RESULTS	20d	Sensitivity analysis results	Present results of sensitivity analyses.	N/A — no sensitivity analyses were performed
RESULTS	21	Reporting biases	Present assessments of risk of bias due to missing results.	N/A — not formally assessed
RESULTS	22	Certainty of evidence	Present assessments of certainty or confidence in the evidence.	N/A — not formally assessed
DISCUSSION	23a	Interpretation	Provide a general interpretation of the results in the context of other evidence.	Discussion
DISCUSSION	23b	Limitations of evidence	Discuss limitations of the included evidence.	Discussion
DISCUSSION	23c	Limitations of review process	Discuss limitations of the review process.	Discussion
DISCUSSION	23d	Implications	Discuss implications for practice, policy, and future research.	Discussion; Conclusions

Section	Item	Topic	Checklist item	Location in revised manuscript
OTHER INFORMATION	24a	Registration and protocol	Provide registration information for the review, including registry and registration number, or state that it was not registered.	Methods, Reporting Guidelines and Registration
OTHER INFORMATION	24b	Protocol access	Indicate where the protocol can be accessed, or state that no protocol is available.	Methods, Reporting Guidelines and Registration
OTHER INFORMATION	24c	Protocol amendments	Describe and explain any amendments to the protocol or state that none apply.	N/A — no prospectively registered protocol
OTHER INFORMATION	25	Support	Describe sources of financial or non-financial support and funder/sponsor role.	Funding statement
OTHER INFORMATION	26	Competing interests	Declare competing interests of review authors.	Conflicts of Interest statement
OTHER INFORMATION	27	Availability of data, code and materials	Report which data, code, and materials are publicly available and where they can be found.	Data Availability Statement

Checklist source: PRISMA 2020 statement and checklist (Page et al., BMJ 2021; PRISMA Executive).
