# Supplementary material for: Endoscopic Hallmarks of Sessile Serrated Lesions: A Systematic Review of an Evolving Concept
Source: Medicina (Kaunas). 2026 Jun 18;62(6):1185. doi: 10.3390/medicina62061185 (PMC13303899; doi:10.3390/medicina62061185)
Supplement: Supplementary file 1 [file medicina-62-01185-s001.zip › medicina-4367128-supplementary-file S2.pdf]

Endoscopic Hallmarks of Sessile Serrated Lesions: A Systematic Review of an Evolving Concept

Supplementary Table S1. Domain-level summary of recurrent QUADAS-related concerns.

QUADAS domain	Concern identified and interpretive impact
Patient selection	Selected rather than consecutive samples and variable exclusion criteria may limit generalizability to routine screening colonoscopy.
Index test interpretation	Blinding between endoscopic assessment and histology was often unclear, which may overestimate the apparent diagnostic value of visual features.
Reference standard	Histology was used as reference standard, but SSL criteria evolved over time; older SSA/P terminology and pre-2019 criteria may contribute to heterogeneity.
Flow and timing	Withdrawals, uninterpretable results, and missing data were incompletely reported, limiting assessment of attrition bias.
Reporting of accuracy	Sensitivity, specificity, predictive values, and interobserver agreement were not consistently reported; reliable pooling or subgroup meta-analysis was therefore not possible.

Supplementary Table S2. QUADAS Assessment of Included Studies

**QUADAS Item**	**Ishigooka 2012**	**Hiraoka 2010**	**Rondagh 2011**	**Singh 2012**	**Boparai 2010**	**Emura 2006**	**Rastogi 2009**	**Tadepalli 2011**	**Kim 2008**	**Sonwalkar 2006**
**Representative patient spectrum**	Yes	Yes	Yes	Yes	Yes	Unclear	Yes	Yes	Unclear	Unclear
**Clear selection criteria**	Yes	Yes	Yes	Yes	Yes	Yes	Yes	Yes	Yes	Yes
**Acceptable reference standard (histology)**	Yes	Yes	Yes	Yes	Yes	Yes	Yes	Yes	Yes	Yes
**Appropriate time interval**	Unclear	Unclear	Yes	Yes	Yes	Unclear	Unclear	Yes	Unclear	Unclear
**Partial verification avoided**	Yes	Yes	Yes	Yes	Yes	Yes	Yes	Yes	Yes	Yes
**Differential verification**	Yes	Yes	Yes	Yes	Yes	Yes	Yes	Yes	Yes	Yes

**avoided**										
**Incorporation bias avoided**	Yes	Yes	Yes	Yes	Yes	Yes	Yes	Yes	Yes	Yes
**Index test described**	Yes	Yes	Yes	Yes	Yes	Yes	Yes	Yes	Yes	Yes
**Reference standard described**	Yes	Yes	Yes	Yes	Yes	Yes	Yes	Yes	Yes	Yes
**Blinding of index test**	Unclear	Unclear	Unclear	Unclear	Unclear	Unclear	Unclear	Yes	Unclear	Unclear
**Blinding of reference standard**	Unclear	Unclear	Yes	Unclear	Unclear	Unclear	Unclear	Yes	Unclear	Unclear
**Clinical data availability**	Yes	Yes	Yes	Yes	Yes	Yes	Yes	Yes	Yes	Yes
**Uninterpretable results reported**	No	No	No	No	No	No	No	No	No	No
**Withdrawals explained**	Unclear	Unclear	Yes	Yes	Yes	Unclear	Unclear	Yes	Unclear	Unclear

Legend: Yes = criterion fulfilled; No = not fulfilled; Unclear = insufficient information reported.
